# The *LRRK2* p.L1795F variant causes Parkinson’s disease in the European population

**DOI:** 10.21203/rs.3.rs-4772543/v1

**Published:** 2024-09-20

**Authors:** Lara M. Lange, Kristin Levine, Susan H. Fox, Connie Marras, Nazish Ahmed, Nicole Kuznetsov, Dan Vitale, Hirotaka Iwaki, Katja Lohmann, Luca Marsili, Alberto J. Espay, Peter Bauer, Christian Beetz, Jessica Martin, Stewart A. Factor, Lenora A. Higginbotham, Honglei Chen, Hampton Leonard, Mike Nalls, Niccolo E. Mencacci, Huw R. Morris, Christine Klein, Cornelis Blauwendraat, Zih-Hua Fang, Global Parkinson’s Genetics Program (GP2)

**Affiliations:** 1 Institute of Neurogenetics, University of Luebeck, Luebeck, Germany; 2 Department of Neurology, University Hospital Schleswig-Holstein, Luebeck, Germany; 3 DataTecnica, Washington, DC, USA; 4 Center for Alzheimer’s and Related Dementias (CARD), National Institute on Aging and National Institute of Neurological Disorders and Stroke, National Institutes of Health, Bethesda, MD, USA; 5 Edmond J. Safra Program in Parkinson’s Disease and the Morton and Gloria Shulman Movement Disorders Clinic, Toronto Western Hospital, University Health Network, University of Toronto, Toronto, Ontario, Canada.; 6 Laboratory of Neurogenetics, National Institute on Aging, National Institutes of Health, Bethesda, MD, USA; 7 University of Cincinnati, Cincinnati, Ohio, USA; 8 CENTOGENE GmbH, Am Strande 7, 18055 Rostock, Germany; 9 Department of Neurology, Emory University School of Medicine, Atlanta, GA, USA; 10 Department of Epidemiology and Biostatistics, Michigan State University, MI, USA; 11 Department of Neurology, Northwestern University Feinberg School of Medicine, Chicago, IL, USA; 12 Department of Clinical and Movement Neurosciences, UCL Queen Square Institute of Neurology, London, UK; 13 UCL Movement Disorders Centre, University College London, London, UK; 14 German Center for Neurodegenerative Diseases (DZNE), Tübingen, Germany

## Abstract

Pathogenic variants in the *LRRK2* gene represent the most common cause of autosomal dominant Parkinson’s disease (PD) worldwide. We identified the *LRRK2* p.L1795F variant in 14 White/European ancestry PD patients, including two families with multiple affected carriers and seven additional affected individuals with familial PD using genotyping and sequencing data from more than 50,000 individuals through GP2, AMP-PD, PDGENEration, and CENTOGENE. All variant carriers were of White/European ancestry, and those with available genotyping data shared a common haplotype. The clinical presentation of p.L1795F carriers resembles that of other *LRRK2* pathogenic variant carriers. Combined with published functional evidence showing strongly enhanced LRRK2 kinase activity, our findings provide conclusive evidence that the *LRRK2* p.L1795F variant is pathogenic. It represents a rare cause of PD in the European population but needs to be included in genetic testing efforts and considered for ongoing gene-specific clinical trials.

## Introduction

Parkinson’s disease (PD) has a complex and multifactorial etiology that includes genetic, environmental, and lifestyle factors, and age^1^. The prevalence of monogenic forms of PD, including pathogenic *GBA1* variants, among relatively unselected PD patients is estimated to be ~15 %^2,3^ with variants in the *LRRK2* gene representing one of the most common causes of autosomal dominant PD, although with reduced penetrance. Since its discovery roughly twenty years ago^4,5^, more than 1,000 different missense variants in the *LRRK2* gene have been identified^6–9^, only a small fraction of which are considered disease-causing. Evaluating newly identified variants in established PD genes can be challenging, but determining pathogenicity is crucial for diagnosis and even more for treatment, particularly now that *LRRK2*-specific clinical trials are underway^10,11^. Key aspects of evaluating newly identified genetic variants are evidence of segregation, absence or very low frequencies in controls, support from *in-silico* prediction tools, and evidence from functional studies showing altered protein function^12,13^.

The *LRRK2* gene encodes the eponymous enzyme Leucine-rich repeat kinase 2 that contains different functional domains, including the N-terminal armadillo, ankyrin, and leucine-rich repeats domains, followed by a C-terminal Roco type GTPase, protein kinase, and WD40 domain. The Roco GTPase domain consists of three subdomains, the ROC GTPase and two scaffolding domains termed COR-A and COR-B^14,15^. Notably, pathogenic variants in *LRRK2* are thought to cause PD through a gain-of-function mechanism leading to increased kinase activity, which impairs endosomal-lysosomal trafficking, promotes neuroinflammation, and affects ciliogenesis in the striatum^14^. Previously, several variants within the interacting ROC, COR-B, and kinase domains have been shown to robustly enhance LRRK2 kinase activity (defined as >1.5-fold above the wild type), measured as the phosphorylation potential of target proteins such as Rab10, supporting their pathogenic role^15^. Amongst those variants was *LRRK2* p.L1795F (chr12:40322386:G:T, hg38), located in the COR-B domain. This variant was reported in a pair of siblings in 2007^16^ and two additional singleton cases with PD were identified in 2016 and 2019^17,18^, nominating it as a possibly causative variant in PD. Moreover, it was recently nominated as a genetic risk factor for PD, with an estimated odds ratio (OR) of 2.5^19^. However, the lack of additional reports of variant carriers and evidence of strong segregation precluded this variant from being considered “pathogenic”.

In our study, we provide conclusive evidence that the *LRRK2* p.L1795F variant is pathogenic by leveraging genome-wide genotyping and whole-genome sequencing data from the Global Parkinson’s Genetics Program (GP2, http://gp2.org/), along with additional data from the Accelerating Medicines Partnership in Parkinson’s Disease (AMP-PD), PDGENEration (PDGENE) and the CENTOGENE database.

## Methods

### Study design and participants

**Figure 1.**
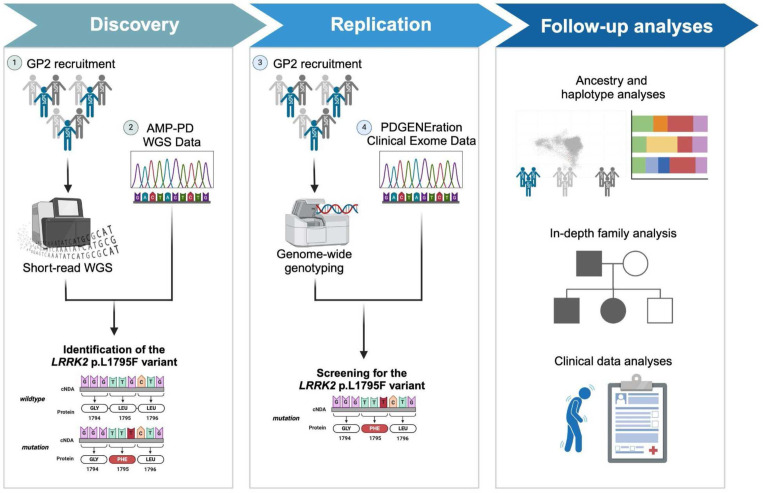
Study design and workflow. Figure created with BioRender.com.

Our study workflow is highlighted in Figure 1. Three sources of data were included in this study (Table 1). First, we used the multi-ancestry whole-genome sequencing and genotyping data from the study participants recruited as part of GP2^20^ as previously described^21,22^. Individual-level demographic and clinical data were obtained from participating principal investigators and publicly available databases (e.g., for Coriell samples included in GP2). Second, we incorporated whole-genome sequencing data from AMP-PD. Participants in this initiative were recruited through multiple studies, including BioFIND, the Harvard Biomarkers Study (HBS), the Lewy Body Dementia Case-Control Cohort (LBD), the Parkinson’s Disease Biomarkers Program (PDBP), the Parkinson’s Progression Markers Initiative (PPMI), the LRRK2 Cohort Consortium (LCC), the Study of Isradipine as a Disease-Modifying Agent in Subjects with Early Parkinson Disease, Phase 3 (STEADY-PD3), and the Study of Urate Elevation in Parkinson’s Disease, Phase 3 (SURE-PD3). Clinical information and genetic samples from participants were obtained with appropriate written consent and local institutional and ethical approvals. Detailed information about these studies is available on the AMP-PD website (https://amp-pd.org) and the respective study websites. Third, we obtained the clinical exome sequencing data from PDGENE^3^, a large multi-center study in North America providing genetic testing and counseling to more than 15,000 participants.

### Whole-genome sequencing (WGS) data

#### AMP-PD

We included 9,974 samples with the sequence alignment data available from BioFIND, HBS, LBD, PDBP, PPMI, STEADY-PD3, and SURE-PD3 cohorts through the AMP-PD release for joint genotyping with the GP2 cohort. Due to the unavailability of sequence alignment data from the LCC cohort, we used AMP-PD release 4 data to screen for potential pathogenic variants in this cohort.

#### GP2

The DNA samples from 5,926 participants were genome sequenced to an average of 30x coverage with 150bp paired-end reads following Illumina’s TruSeq PCR-free library preparation protocol. We followed the same functional equivalence pipeline^23^ as AMP-PD to produce the sequence alignment against the GRCh38DH reference genome.

We used DeepVariant v.1.6.1^24^ to generate the single-sample variant calls for a total of 15,900 samples in GP2 and AMP-PD and performed joint-genotyping using GLnexus v1.4.3 with the preset DeepVariant WGS configuration^25^. We set genotypes to be missing after variant quality control defined as genotype quality >=20, read depth >=10, and heterozygous allele balance between 0.2 and 0.8, and retained high-quality variants with a call rate > 0.95 after quality control. After the sample quality control following the quality metrics defined by AMP-PD^26^, we retained 15,752 samples (AMP-PD and GP2 combined) for the downstream analyses (Supplementary Table 1). Variant annotation was performed with Ensembl Variant Effect Predictor v111^27^. We used KING v.2.3.0^28^ to infer relatedness up to the second-degree relatives to confirm the known relationships and identify cryptic familial relationships. Genetic ancestry was determined using GenoTools v1.2.3 with the default settings^29^.

### Genome-wide genotyping with the Neurobooster Array (GP2)

We screened the genotyping data published as part of GP2’s Data Release 7^30^ (Supplementary Table 2). Genotyping was performed by GP2 using the NeuroBooster Array^31^ (NBA; v.1.0, Illumina, San Diego, CA). Raw genotyping data underwent quality control and genetic ancestry prediction using GenoTools v1.2.3 with the default settings^29^. The *LRRK2* p.L1795F variant was directly genotyped using NBA, and the quality of genotype calls was assessed by examining the signal intensity plots.

### Clinical exome sequencing (PDGENEration)

We included 9,759 samples with clinical exome data available from PDGENE^3^. The sequence data processing followed the same pipeline of WGS data as mentioned above. We performed joint-genotyping using GLnexus v1.4.3 with the preset DeepVariant WES configuration and followed the same criteria for sample and variant quality control as for the WGS data.

### Querying additional databases (CENTOGENE)

We queried the CENTOGENE proprietary Databank CentoMD^®^^32^ to identify potential additional variant carriers. CENTOGENE is a globally operating genetic diagnostic lab. Genetic data included in this manuscript was generated by exon-wise PCR amplification followed by Sanger sequencing.

### Statistical analyses

To estimate the allele frequency of *LRRK2* p.L1795F variant in multi-ancestral populations, we analyzed the GP2 genotyping data, the largest available dataset in this study. We excluded related individuals and samples from targeted recruitment, such as *LRRK2* and *GBA1* variant carriers within specific efforts of PPMI and LCC. Subsequently, we performed an association analysis of this variant with PD using the European population. We fitted the logistic regression model with PD status as binary outcome variable and the covariates as the genotype of *LRRK2* p.L1795F variant, sex, age, family history, and the first six principal components to account for the population stratification. For cases, age at onset (AAO) or age at diagnosis was used, while for controls, age at sampling was used. Additionally, we merged GP2 genotyping data with the combined AMP-PD and GP2 WGS data, resulting in a cohort of 23,276 PD cases of European ancestry after excluding duplicated, related, and targeted recruitment samples as mentioned above. This allowed us to compare the carrier distribution between PD cases and non-Finnish European population from the Genome Aggregation Database (gnomAD v4.1) as external population controls using Fisher’s exact test. We excluded the PDGENE clinical exome data from this analysis as we could not estimate the genetic ancestry in the same manner as with the other datasets. The *P* value ≤0.05 was considered statistically significant for all the analyses.

To determine if carriers of the *LRRK2* p.L1795F variant shared recent common ancestry, we phased the genotyping data from chromosome 12 in the European population using Beagle 5.4 with default settings^33^ and searched for identical-by-descent (IBD) segments with the length ≥2 cM shared across the carriers using hap-ibd with default setting^34^.

## Results

### Identification of the *LRRK2* p.L1795F variant segregating with disease in a family

**Figure 2.**
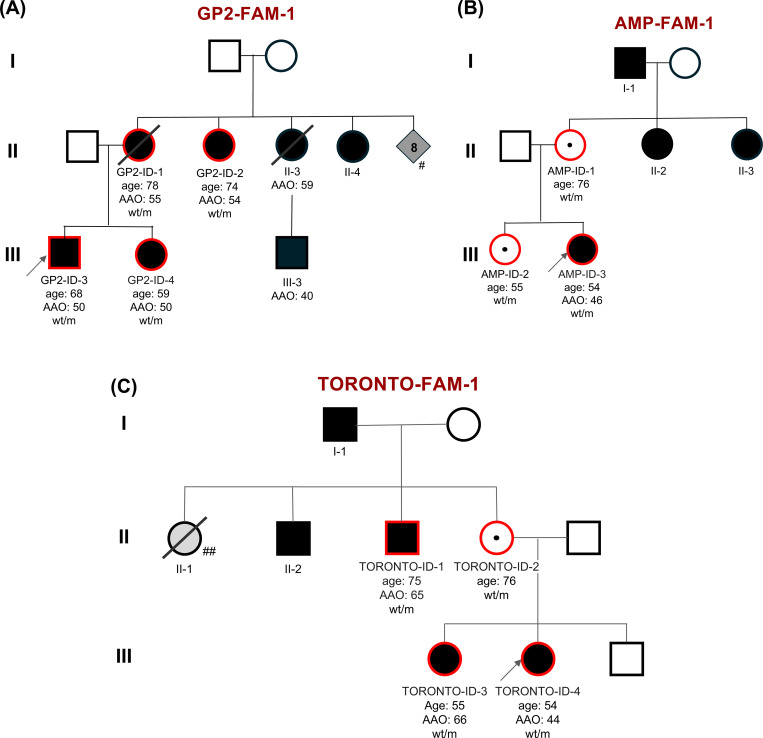
Pedigree of Family GP2-FAM-1 (A), AMP-FAM-1 (B), and TORONTO-FAM-1 (C) with the *LRRK2* p.L1795F variant. The pedigrees were drawn based on reported family history and may be incomplete. The index cases are indicated with arrows. Affected individuals are indicated by black symbols: circles (female) and squares (male). Diamond is where sex is undefined. Unaffected individuals are indicated by open symbols. Unaffected variant carriers are indicated by open symbols with a dot in the middle. A diagonal line indicates deceased individuals. Red circle indicates individuals with genetic data available (WGS data for GP2-FAM-1 and AMP-FAM-1, single gene testing for TORONTO-FAM-1). Heterozygous mutant (m) and wild-type (wt) genotypes are indicated with corresponding age at the sample collection (age) and age at motor symptom onset (if known; AAO). (A) The mother of GP2-FAM-1 index was reported to have eight additional siblings (#), several of whom are clinically affected with PD; however, no detailed family history is available for these relatives. (C) One maternal aunt (II-1) of the TORONTO-FAM-1 index was reported to have had Alzheimer’s disease (##).

Our discovery cohort consisted of 16,351 individuals from GP2 and AMP-PD with WGS data, including 15,752 samples from the joint-genotyping sample set and 599 samples from the LCC cohort from AMP-PD release 4 (Table 1). Searching for recurrent rare variants, we identified nine carriers of the *LRRK2* p.L1795F variant (ENST00000298910:c.5385G>T; chr12:40322386:G:T; Supplementary Figures 1-6). Of these carriers, we identified two families based on kinship inference (Figure 2). The larger family (GP2-FAM-1) consisted of four affected individuals showing segregation of this variant with PD. The second family (AMP-FAM-1) consisted of three variant carriers, including one clinically affected with PD, while the other two were reported as asymptomatic at age 55 and 76 years. The remaining two carriers were singleton cases with familial PD. Next, we screened the genotyping data of 54,153 affected and unaffected individuals generated within GP2 and identified three additional variant carriers, all clinically affected with PD (Supplementary Figure 7). Further, screening the clinical exome data from 9,759 individuals available from PDGENE resulted in one additional variant carrier (Supplementary Figure 8). Finally, querying the CENTOGENE proprietary Databank CentoMD^®^^32^, we identified another family with four individuals carrying the *LRRK2* p.L1795F variant, three of whom were clinically affected with PD and one being an asymptomatic carrier. In total, we identified 17 individuals carrying this variant across all the datasets, of which 14 had PD and three were asymptomatic.

### Evaluation of the *LRRK2* p.L1795F variant

The p.L1795F (c.5385G>T) missense variant is rare and confined to European populations in several investigated databases, including gnomAD v4.1 (MAF_Europeans(non-Finnish)_=0.000001695) and the Regeneron Genetics Center Million Exome Variant Browser^35^ (RGC-ME, MAF_Europeans_=0.000009515). In comparison, it was not present in the UK Biobank^36^ 500K genomes. Evaluation using various *in-silico* prediction tools and databases presents conflicting results. ClinVar, Varsome, and Franklin (the latter two based on the ACMG criteria^12^) categorize this variant as a variant of uncertain significance. Notably, Varsome and Franklin do not take the existing functional evidence^15^ into account. Furthermore, this variant is currently not included in the list of genetic variants reported to the clinician or the participant in PDGENE^3^. MutationTaster predicts the variant to be disease-causing, and the leucine at position 1795 is conserved across different species (Supplementary Figure 9). In contrast, other *in-silico* tools for predicting missense pathogenicity did not support pathogenicity following ACMG recommended thresholds^37^, including CADD (19.94) and REVEL (0.638), with the exception of VEST4 (0.928). Finally, this variant is located in the COR-B subdomain of the C-terminal Roco GTPase domain and has previously been shown to strongly increase LRRK2 kinase activity^15^.

### Allele frequency of *LRRK2* p.L1795F in multi-ancestral populations and the founder effect

All identified *LRRK2* p.L1795F carriers in this study were of European ancestry, whereas the variant was absent in other ancestral populations (n=15,316) within the GP2 genotyping cohort. It had an allele frequency of 0.00012 among PD cases (5 heterozygous carriers and 20,812 noncarriers) while being absent in controls (n=9,032) in the European population of the GP2 genotyping cohort (Table 2). The logistic regression analysis using the European population of the GP2 genotyping cohort did not reveal a significant association between this variant and PD, possibly due to insufficient controls available in the dataset (*P*>0.8, Supplementary Table 3). However, when comparing the carrier distribution between PD cases from the combined genotyping and WGS dataset (6 heterozygous carriers and 23,270 noncarriers) with the non-Finnish European population from Genome Aggregation Database (gnomAD v4.1) as external population controls (2 heterozygous carriers and 589,826 noncarriers), this variant showed a significant association with PD (*P*<7.84e-08, two-tailed, Fisher’s exact test).

**Figure 3.**
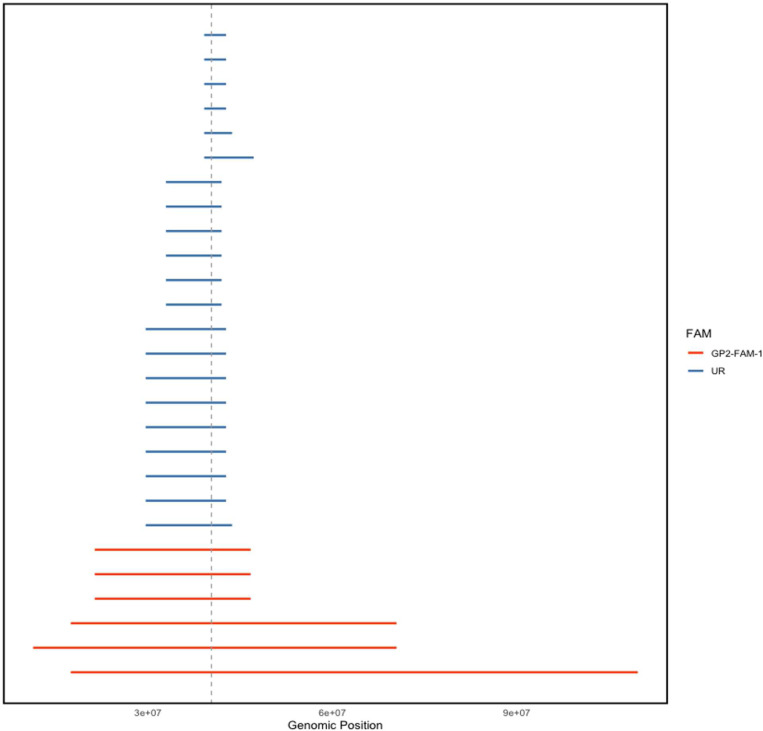
Overlapping identity-by-descent segments spanning *LRRK2* p.L1795F variant among the variant carriers with genotyping data. Each line represents an IBD segment inferred between a unique pair. IBD segments have been coloured according to whether both individuals within a pair belong to the same family (GP2-FAM-1) or are otherwise considered unrelated (UR). Vertical grey line represents the genomic position of *LRRK2* p.L1795F.

Given this variant was observed only in the European population from the GP2 genotyping cohort, we searched for the overlapping IBD segments among the variant carriers using the genotyping data (Figure 3). The median length of an IBD segment over *LRRK2* in these individuals was 7.05 cM (range: 2.1–96.3 cM, Supplementary Table 4). All genotyped carriers shared a core haplotype of 2.825 Mbp at this locus (Supplementary Table 5), suggesting that the p.L1795F variant descended from a common founder.

### Clinical features of identified *LRRK2* p.L1795F variant carriers

The demographic and clinical details of all 17 identified variant carriers of White/European ancestry, including 14 affected and three unaffected individuals, are displayed in Table 3. More than two-thirds were females (70.6 %; n=12/17). All affected and unaffected carriers had a positive family history of PD. Ages of motor symptom onset in affected individuals ranged from 36 to 66 years. The median AAO was 54.5 years (interquartile range 47–60 years). The asymptomatic carriers were 55, 76 and 76 years old, respectively, at the time of sample collection. Based on the available clinical data, the majority of affected individuals had classical PD with an asymmetric onset of symptoms and a good response to dopaminergic medication, and without obvious atypical signs suggestive of other diagnoses (missing data for up to 30%); detailed data on non-motor symptoms and neuropsychiatric comorbidities were scarce. Cognition was reported to be unaffected in the majority of affected carriers with good scores in cognition tests (including Montreal Cognitive Assessment [MoCA] and Mini Mental State Examination [MMSE]); however, one clinically affected individual had significant cognitive impairment (MoCA score of 17/30 points) and one unaffected carrier also showed some cognitive deficits (MoCA score of 23/30 points). The characteristics of the individuals from the three identified families will be reported in more detail.

#### Family GP2-FAM-1

Family GP2-FAM-1 is of European ancestry with Ukrainian and Polish origin. Seven individuals are known to be clinically affected by PD, including the index patient (GP2-ID-3), his sister, mother, three maternal aunts, and a maternal cousin, consistent with autosomal dominant inheritance (Figure 2). Further, additional maternal aunts and uncles were reported to have PD but a detailed history was not available. We identified the *LRRK2* p.L1795F variant segregating within all four tested family members from both NBA and WGS data. No unaffected family samples were available. Screening variants segregating within this family from the WGS data did not reveal any other potential causal variants, including known pathogenic variants in the established dominant PD genes *SNCA* and *VPS35* as well as other variants in *LRRK2* and pathogenic *GBA1* variants.

All family members with available data were reported to have bradykinesia, rigidity, resting and action tremor, and motor symptoms that were responsive to dopaminergic treatment. Disease progression was mild to moderate in three of four individuals with low to moderate UPDRS (part III) motor scores and a Hoehn & Yahr stage 2 after 9+ years of disease duration. Only one individual, GP2-ID-1 (deceased), seemed to have had a more progressive disease course with a high UPDRS (part III) motor score and Hoehn & Yahr stage 5, though over a disease duration of more than 20 years. Neuropsychiatric comorbidities or severe autonomic features were not reported in those with available data. Cognition was unaffected in all family members. All but one affected family member, including those without genetic testing, had an AAO in their fifties (ranging from 50 to 59 years), and only one individual had a lower AAO of 40 years. Videos of individuals GP3-ID-3 and GP2-ID-4 are available in the supplementary materials.

#### Family AMP-FAM-1

Family AMP-FAM-1 included three individuals available for genetic testing, all of whom carried the p.L1795F variant. The index case was clinically affected by PD, while her sister and mother were both asymptomatic. The family history of PD was strongly positive, with multiple additional affected family members, including two maternal aunts and the maternal grandfather of the index, suggesting autosomal dominant inheritance with reduced penetrance (Figure 3). The index patient had a reported AAO of 46 years and a very low UPDRS (part III) motor score, indicating a rather mild disease course. Clinical details for the additional affected family members were unavailable. The two asymptomatic carriers were 55 and 76 years old at sample collection and showed no signs of PD. We did not identify other potential disease-causing variants in this family by WGS.

#### Family TORONTO-FAM-1

Family TORONTO-FAM-1 included four individuals available for genetic testing, and all four carried the *LRRK2* p.L1795F variant. The index case as well as her sister and a maternal uncle were clinically affected with PD whereas the mother of both siblings was an unaffected carrier. There were additional family members clinically affected with PD, including another maternal uncle and the maternal grandfather, both of which were unavailable for genetic testing within this study (Figure 2).

The reported AAO of the index case was 44 years and thereby younger than for the other two tested family members, which were 65 and 66, respectively. The index patient and her sister have been followed up for almost 12 years, whereas the other two individuals (TORONTO-ID-1 and TORONTO-ID-2) were only clinically assessed once in 2012. Notably, the index patient had a more progressive PD disease course than her sister, as indicated by a higher UPDRS motor score of 43 points and Hoehn & Yahr stage 3, compared to only 7 points in the UPDRS (part III) in her sister. All three affected individuals had a diagnosis of classical PD without any atypical features; however, one individual (TORONTO-ID-1) had significant cognitive impairment with a low MoCA score of only 17 out of 30 points. Interestingly, also the unaffected carrier showed some cognitive impairment (MoCA score of 23/30 points) but no motor symptoms of PD.

## Discussion

Our study was carried out under the umbrella of GP2, a large international collaborative effort aimed at better understanding the genetic architecture of PD at a global scale by generating large-scale genetic data from diverse ancestries. Additionally, we leveraged data from AMP-PD and PDGENE and queried the CENTOGENE database. To investigate monogenic causes of the disease, we screened WGS data from our discovery cohort for recurrent rare variants. We identified the *LRRK2* p.L1795F variant segregating with the disease in four members of a large European ancestry family, with multiple additional affected family members not available for genetic analyses. Moreover, the variant was identified in a second family with one affected and two asymptomatic carriers, alongside multiple affected family members not available for genetic analyses. Further, we identified four affected carriers by analyzing additional datasets, including NBA genotyping data from GP2 and clinical exome data from PDGENE. Finally, we identified another family with 3 affected individuals and one unaffected carrier by querying the CENTOGENE database. All identified variant carriers in this study were of non-Ashkenazi Jewish, non-Finnish European ancestry and had a strongly positive family history with at least one but more often multiple additional affected family members. A previous rare-variant association analysis further supports the role of p.L1795F in PD pathogenesis, identifying it as a genetic risk factor with an estimated OR of 2.5^19^. However, it should be noted that the number of identified carriers was quite small, likely resulting in an underestimation of the actual OR. Most importantly, our findings provide family segregation evidence missing from the previous reports^16–18^. Furthermore, the previously reported increase in kinase activity of this variant aligns with the disease mechanism established for several pathogenic *LRRK2* variants^15^. When applying the ACMG criteria^12^ in light of our findings, the variant can now be classified as pathogenic based on: i) the very low frequency in population databases (PM2), ii) established functional studies supporting a damaging effect consistent with the established disease mechanism (PS3), iii) observation of the variant in multiple unrelated individuals with the same phenotype (no specific criterion, may be considered as moderate evidence), and iv) strong evidence of segregation (at least PP1, based on our findings upgraded to strong evident by segregation in three families with two generations of family members each). Taken together, we thereby propose the *LRRK2* p.L1795F variant to be considered pathogenic and causative of PD.

Interestingly, the *LRRK2* p.L1795F variant had an estimated allele frequency of 8.37×10^−5^ (5 observations in 59,698 alleles) in the European population and was absent from all other ancestral populations in the GP2 genotyping cohort. This finding was consistent with several public frequency databases, such as gnomAD v4.1 and RGC-ME, contrasting with the *LRRK2* p.G2019S variant. Globally, the *LRRK2* p.G2019S variant is the most common and well-studied genetic cause of PD. Due to independent founder effects^38–40^, the highest frequencies of this variant were observed in the Ashkenazi Jewish population^41^, ranging from 10% in sporadic to 26% in familial PD, and Arab-Berber populations, ranging from 30% in sporadic to 41% in familial PD^42^. The variant was also commonly reported in individuals of Portuguese, Brazilian, Spanish, and Italian ancestry but is much rarer in individuals of other European, Asian, or Indian descent^43^. In comparison, only four individuals carrying the p.L1795F were reported^16–18^, and two additional carriers were identified through AMP-PD^6,19^. To our knowledge, we provide the largest number of p.L1795F variant carriers thus far, including 14 carriers clinically affected with PD and three asymptomatic carriers. The available data on AAO and family pedigrees from these previously reported carriers^16–18^ do not align with our data, making an overlap of individuals between the different studies unlikely. Including those reported in the literature, this brings the total to 18 clinically affected carriers of European ancestry. In our GP2 genotyping cohort, the observed allele frequency of the p.L1795F variant among affected European individuals (n=20,817) was 0.00012, while the allele frequency for the p.G2019S variant was 0.003266 (Table 2). This indicates that p.G2019S is a more common cause of PD in the European population compared to p.L1795F. However, we acknowledge that the overall number of p.L1795F carriers is still limited, and higher frequencies might be observed in specific European subpopulations. Our haplotype analysis further supports this hypothesis, where all genotyped carriers shared a core haplotype of 2.83 Mbp. We were able to determine the geographical origin of only one family of carriers in this study, which was of Ukrainian and Polish descent. Additionally, this variant is more prevalent in Northern Europe according to ancestry estimates of the carriers from RGC-ME but was not found in the 500K genomes of the UK Biobank. Consequently, investigating the *LRRK2* p.L1795F variant within the Central-Eastern European population could offer additional insights into a possible founder event.

Comparing the clinical phenotypes of the p.L1795F carriers with those of other pathogenic *LRRK2* variants, particularly p.G2019S^13^, revealed similarities among them and with idiopathic PD (iPD). LRRK2-PD is clinically indistinguishable from iPD on an individual level. Most individuals with LRRK2-PD, including p.L1795F carriers, exhibit a classic PD phenotype with asymmetric disease onset and display all the cardinal motor signs of PD with a good response to dopaminergic treatment. Atypical presentations have been described in single cases but are overall rare^43^. Furthermore, the AAO was comparable between p.L1795F carriers and other LRRK2-PD genetic subtypes. Most individuals exhibited first motor symptoms in their 50s and 60s (53% in LRRK2-PD overall^13^ and 70 % of all known p.L1795F carriers including our study). However, a broader range of age at onset has been described, spanning from 20 to 95 years for LRRK2-PD overall^13^ and from 25 to 66 years for p.L1795F carriers. Non-motor features and neuropsychiatric comorbidities haven’t been specifically reported for the majority of p.L1795F carriers, but the overall data is limited, making it difficult to draw meaningful conclusions. While group differences in clinical phenotypes among *LRRK2* variants may exist^43^, they do not enable meaningful genotype-phenotype correlations at an individual level. Overall, the p.L1795F phenotype aligns well with the general characteristics of LRRK2-PD and appears comparable to other *LRRK2* variants with cautious interpretation given the limited number of identified carriers. The most significant differences between the genetic subtypes are their ancestral and geographical variability.

Notably, we identified three asymptomatic p.L1795F carriers from two different families who might still develop PD symptoms later in life. However, this seems unlikely for at least two individuals, who were 76 years old at the most recent follow-up, given that the oldest reported age at onset (AAO) for the affected p.L1795F carriers is currently 66 years^16^. Alternatively, reduced penetrance, a common phenomenon in monogenic forms of PD, including other pathogenic *LRRK2* variants, might explain the finding. All three asymptomatic p.L1795F carriers were first-degree relatives of an affected carrier. Additionally, several other family members with PD were reported in these families, suggesting that these affected members might also carry the same variant. However, they were not available for genetic testing in this study. *LRRK2* penetrance depends on age^44^, environmental and lifestyle factors^45^, ancestral background^46,47^, and the specific variant as well as additional genetic modifiers^48–50^. For example, penetrance of the most common p.G2019S variant is estimated at around 25–30% in the Ashkenazi Jewish population and up to 42% in non-Jewish individuals by the age of 80 years, and 45% in the North African Berber population over their life course^43^. However, the current data on *LRRK2* p.L1795F is still limited, and the number of tested affected and unaffected family members is too low to estimate the penetrance for this variant accurately.

In conclusion, this is the first study providing evidence of the *LRRK2* p.L1795F variant segregating with disease in large multiplex families. Taken together with published functional data, showing strongly enhanced LRRK2 kinase activity, our findings support the *LRRK2* p.L1795F variant to be considered pathogenic. Our study demonstrates that large-scale studies can be helpful to identify novel rare causes of PD but also to re-evaluate previously identified variants by providing additional evidence of pathogenicity through an increased number of variant carriers and segregation. We, therefore, propose *LRRK2* p.L1795F as a cause of PD, especially in the European population. Including this variant in the genetic screening of PD patients may be beneficial for the variant carriers to be included in ongoing gene-specific clinical trials.

## Figures and Tables

**Table 1:** Overview of the investigated cohorts.

Cohort	Discovery		Replication	
	GP2	AMP-PD [3]	GP2	PDGENE
**Data type**	WGS	WGS	NBA	CES
**Total number of samples**	5,796	9,956 (599)	54,180	9,759
**PD cases**	5,283	3,442	28,729	9,759
**Other phenotypes** [1]	161	2,903	15,834	NA
**Controls** [2]	342	4,210	9,617	NA

AMP-PD = Accelerating Medicines Partnership Parkinson’s disease, GP2 = Global Parkinson’s Genetics Program, NA = not available, NBA = NeuroBooster Array, PD = Parkinson’s disease, PD GENE = PD GENEration study, CES = clinical-exome sequencing, WGS = whole-genome sequencing

[1] Other phenotypes include atypical parkinsonism, e.g., progressive supranuclear palsy (PSP), multi system atrophy (MSA), corticobasal degeneration/syndrome (CBD/CBS), and dementia with Lewy bodies (DLB), as well as prodromal PD.

[2] Controls include asymptomatic carriers of known pathogenic variants.

[3] Joint-genotyping using the 9,956 samples from BioFIND, Harvard Biomarkers Study (HBS), Lewy body dementia case-control cohort (LBD), Parkinson’s disease Biomarkers Program (PDBP), Parkinson’s Progression Markers Initiative (PPMI), Study of Isradipine as a Disease-modifying Agent in Subjects With Early Parkinson Disease, Phase 3 (STEADY-PD3), Study of Urate Elevation in Parkinson’s Disease, Phase 3 (SURE-PD3), and Postmortem Cohort.

AMP-PD release 4 was used to screen for potential pathogenic variants for the 599 samples from the LRRK2 Cohort Consortium (LCC).

**Table 2:** Frequency of the *LRRK2* p.L1795F and p.G2019S variants across ancestries in the GP2 genotyping cohort.

Variant	Ancestry	AF in cases	AF in controls	Number of alleles in cases (AN_case)	Number of alleles in controls (AN_control)
**chr12:40322386:G:T (*LRRK2* p.L1795F)**	EUR	0.0001201	0	41634	18064
**chr12:40340400:G:A (*LRRK2* p.G2019S)**	AAC	0	0.0006281	568	1592
	AFR	0	0	1876	3252
	AJ	0.07081	0.01098	2556	820
	AMR	0.01339	0.003247	896	308
	CAH	0.006783	0.003436	1032	582
	CAS	0	0	1104	688
	EAS	0	0	5122	4752
	EUR	0.003266	0.000166	41636	18074
	FIN	0	0	192	14
	MDE	0.02805	0	606	446
	SAS	0	0	732	412

AF = Allele frequency, AAC= African admixed, AFR= African, AJ = Ashkenazi Jewish, AMR = Latino and Indigenous people of the Americas, CAH = Complex Admixture History, CAS= Central Asian, EAS = East Asian, EUR = European, FIN = Finnish, MDE = Middle Eastern, SAS = South Asian

*LRRK2*: ENST00000298910.12; ENSP00000298910.7

**Table 3:** Demographic and clinical characteristics of identified *LRRK2* p.L1795F variant carriers.

Cohort	GP2								AMP-PD				PD GENE	TORONTO			
Family ID	GP2-FAM-1				NA	NA	NA	NA	AMP-FAM-1			NA	NA	TORONTO-FAM-1			
Sample ID	GP2-ID-1	GP2-ID-2	GP2-ID-3	GP2-ID-4	GP2-ID-5	GP2-ID-6	GP2-ID-7	GP2-ID-8	AMP-ID-1	AMP-ID-2	AMP-ID-3	AMP-ID-4	PDGENE-ID-1	TORONTO-ID-1	TORONTO-ID-2	TORONTO-ID-3	TORONTO-ID-4
Genetic method	NBA, WGS	NBA, WGS	NBA, WGS	NBA, WGS	NBA, WGS	NBA	NBA	NBA	WGS	WGS	WGS	WGS	CES	Single gene testing (*LRRK2*)			
**Demographics**																	
Gender	Female	Female	Male	Female	Male	Male	Female	Male	Female	Female	Female	Female	Female	Male	Female	Female	Female
Genetic ancestry	EUR	EUR	EUR	EUR	EUR	EUR	EUR	EUR	EUR	EUR	EUR	EUR	EUR	White	White	White	White
Age at sample collection	78	74	68	59	42	72	62	76	76	55	54	69	57	75	76	55	54
Family history of PD	yes	yes	yes	yes	yes	yes	yes	yes	yes	yes	yes	yes	yes	yes	yes	yes	yes
Family history details	two children, three sisters, one nephew, several aunts and uncles	three sisters, one niece and two nephews, several aunts and uncles	sister, mother, three maternal aunts	brother, mother, three maternal aunts	aunt, two great uncles	brother, mother	mother, sister	mother	father, two siblings, child	sibling, maternal grandparent, maternal aunt	maternal grandprarent, two maternal aunts	mother	maternal grandmother	father, two siblings, two nieces	father, two siblings, two children	sibling, mother, two maternal uncles, maternal grandfather	sibling, mother, two maternal uncles, maternal grandfather
**Clinical data**																	
Diagnosis	PD	PD	PD	PD	PD	PD	PD	PD	Control*	Control*	PD*	PD	PD	PD	Control**	PD	PD
Age at motor symptom onset	55	54	50	50	36	60	57	55	NA	NA	46	65	47	65	NA	66	44
Bradykinesia	+	+	+	+	+	+	+	+	NA	NA	+	+	+	+	-	+	+
Rigidity	+	+	+	+	+	-	+	+	NA	NA	+	+	+	-	-	+	+
Resting Tremor	+	+	+	+	+	+	-	+	NA	NA	+	+	-	+	-	-	-
Action/Kinetic Tremor	+	+	+	+	-	+	+	NA	NA	NA	-	+	-	-	-	+	+
Postural Instability	+	+	-	+	+	-	+	+	NA	NA	-	-	-	-	-	-	+
Gait Disturbance	+	+	-	+	+	-	-	NA	NA	NA	-	+	-	-	-	-	+
Asymmetric onset of symptoms	+	+	+	+	+	+	+	NA	NA	NA	+	NA	+	+	-	-	+
Responsive to dopaminergic medication	+	+	+	+	+	+	+	NA	NA	NA	+	NA	+	NA	NA	NA	+
Fluctuations	NA	NA	+	+	-	NA	NA	NA	NA	NA	+	NA	+	-	-	-	+
UPDRS Part III (motor score)	70	NA	10	22	24	6	11	NA	NA	NA	3	32	6	6	0	7	43
Hoehn & Yahr	5	2	2	2	2	1	1.5	NA	NA	NA	2	2	2	1	0	0	3
Cognition	MMSE 29	MMSE 29	MMSE 30	MMSE 30	MMSE 30	MMSE 30	MMSE 30	NA	NA	NA	MoCA 28	NA	-	MoCA 17	MoCA 23	MoCA 29	MoCA 28
Neuropsychiatric features	NA	NA	-	-	NA	NA	NA	NA	NA	NA	NA	NA	-	NA	NA	-	+
Dysautonomia	-	-	-	constipation	-	-	-	NA	NA	NA	NA	NA	-	-	-	-	-
Atypical Features or signs suggestive of other diagnosis (#)	history of head trauma with loss of conciousness	-	-	-	history of head trauma with loss of conciousness	-	-	NA	NA	NA	NA	NA	-	-	-	-	-

+ present;

- absent

EUR = European, MMSE = Mini Mental State Examination, MOCA = Montreal Cognitive Assessment, NA = Not available or applicable, NBA = NeuroBooster Array, PD = Parkinson’s disease, CES = clinical-exome sequencing, WGS = Whole-genome sequencing

* Individuals were recruited through the LCC as “Genetically enriched” study arm.

** Recruited as unaffected family member, not population control.

(#) These include: history of strokes or stepwise deterioration, history of head injury with loss of consciousness, history of encephalitis, Oculogyric crisis, neuroleptic treatment at time of symptom onset, sustained remission, gaze palsy, Cerebellar signs (other than activation tremor), Fluctuations, hallucinations, dysautonomia, Memory loss, axial rigidity, Other

## Data Availability

GP2 partnered with the online cloud computing platform Accelerating Medicines Partnership - Parkinson’s Disease (AMP PD; https://amp-pd.org) to share data generated by GP2. Anonymized data can be shared upon request and qualified researchers are encouraged to apply for direct access to the data through AMP PD.
